# Properties and Application of Cell-Free DNA as a Clinical Biomarker

**DOI:** 10.3390/ijms22179110

**Published:** 2021-08-24

**Authors:** Felipe Silva de Miranda, Valério Garrone Barauna, Leandro dos Santos, Gustavo Costa, Paula Frizera Vassallo, Luciene Cristina Gastalho Campos

**Affiliations:** 1 Post Graduation Program in Biology and Biotechnology of Microorganisms, State University of Santa Cruz, Ilhéus 45662-900, Bahia, Brazil; felipemiranda2004@hotmail.com; 2 Department of Biological Science, State University of Santa Cruz, Ilhéus 45662-900, Bahia, Brazil; 3 Laboratory of Applied Pathology and Genetics, State University of Santa Cruz, Ilhéus 45662-900, Bahia, Brazil; 4 Post Graduation Program in Health Sciences, State University of Santa Cruz, Ilhéus 45662-900, Bahia, Brazil; barauna2@gmail.com; 5 Molecular Physiology Laboratory of Exercise Science, Federal University of Espírito Santo, Vitória 29075-910, Espírito Santo, Brazil; 6 Post Graduation Program in Physiological Sciences, Federal University of Espírito Santo, Vitória 29075-910, Espírito Santo, Brazil; gustavo_ufes@hotmail.com (G.C.); pfvassallo1@gmail.com (P.F.V.); 7 Academic Unit of Serra Talhada, Rural Federal University of Pernambuco, Serra Talhada 56909-535, Pernambuco, Brazil; leandro.santos.79@gmail.com; 8 Clinical Hospital, Federal University of Minas Gerais, Belo Horizonte 30130-100, Minas Gerais, Brazil

**Keywords:** cfDNA, biomarker, endothelial dysfunction, vascular damage, circulating nucleotide

## Abstract

Biomarkers are valuable tools in clinical practice. In 2001, the National Institutes of Health (NIH) standardized the definition of a biomarker as a characteristic that is objectively measured and evaluated as an indicator of normal biological processes, pathogenic processes, or pharmacological responses to a therapeutic intervention. A biomarker has clinical relevance when it presents precision, standardization and reproducibility, suitability to the patient, straightforward interpretation by clinicians, and high sensitivity and/or specificity by the parameter it proposes to identify. Thus, serum biomarkers should have advantages related to the simplicity of the procedures and to the fact that venous blood collection is commonplace in clinical practice. We described the potentiality of cfDNA as a general clinical biomarker and focused on endothelial dysfunction. Circulating cell-free DNA (cfDNA) refers to extracellular DNA present in body fluid that may be derived from both normal and diseased cells. An increasing number of studies demonstrate the potential use of cfDNA as a noninvasive biomarker to determine physiologic and pathologic conditions. However, although still scarce, increasing evidence has been reported regarding using cfDNA in cardiovascular diseases. Here, we have reviewed the history of cfDNA, its source, molecular features, and release mechanism. We also show recent studies that have investigated cfDNA as a possible marker of endothelial damage in clinical settings. In the cardiovascular system, the studies are quite new, and although interesting, stronger evidence is still needed. However, some drawbacks in cfDNA methodologies should be overcome before its recommendation as a biomarker in the clinical setting.

## 1. cfDNA—Historical Perspective

Circulating cell-free DNA (cfDNA) are extracellular fragments of DNA present in body fluid that may be derived from both normal and diseased cells [1]. cfDNA molecules were discovered in the human circulatory system in 1948 by Mandel and Metais [2]. Seventeen years later, in 1965, Bendich et al. [3] hypothesized that cancer-derived cfDNA was a determining factor in oncogenesis, specifically favoring the metastatic spread of cancer. Certainly, due to a lack of knowledge regarding its composition, function, and biological and evolutionary origins, cfDNA did not receive significant attention for the next 55 years after its discovery. In 1966, the link to disease state was first noted when Tan et al. [4] identified high levels of cfDNA in the blood of systemic lupus erythematosus.

Ten years later, Leon et al. [5] demonstrated, through radio-immunochemistry assay, that cancer patients featured a higher level of cfDNA than normal subjects. Furthermore, cfDNA levels decreased with the anticancer therapy success. However, because of technological limitations, only 12 years later, in 1989, Stroun et al. [6] demonstrated the first experimental evidence that identified the origin of cfDNA in cancer patients. The authors observed the double-strand instability specific to the tumor DNA in the cfDNA, and thus, it became also to be called ctDNA (circulating-tumoral DNA).

The progress of molecular biology techniques in the 1990s associated with the human genome project development allowed a more direct demonstration of this tumor origin. In 1994, Vasioukhin et al. [7] and Sorenson et al. [8] showed tumor-specific (N-RAS) mutations in the plasma samples of patients with pancreatic adenocarcinoma and acute myelogenous leukemia. In parallel, other specific analyses of cfDNA had become of interest in a clinical domain. In 1997, Lo et al. [9] identified fetal cfDNA in maternal plasma and serum. These observations opened a range of opportunities suggesting maternal plasma/serum DNA as a useful source for noninvasive prenatal diagnosis of genetic disorders in obstetrics.

In the following years, new studies showed evidence of cfDNA methylation as an epigenetic biomarker. Simultaneously, in 1999, two groups performed for the first time the cfDNA methylation evaluation in humans. Esteller et al. [10] detected aberrant promoter hypermethylation of tumor suppressor genes in cfDNA from non-small cell lung cancer patients, and Wong et al. [11] detected aberrant methylation of the p16 gene in the cfDNA of hepatocellular carcinoma patients. Both showed for the first time that the presence of aberrant promoter methylation could be detected in the peripheral circulation of cancer patients with hepatocellular carcinoma.

Although previous studies have shown that tumor-specific DNA and fetal DNA are present in plasma and serum from patients with cancer and pregnant women, the first evidence of mitochondrial cfDNA (mt-cfDNA) in plasma and serum was shown in 2000 by Zhong et al. [12]. At this moment, the nomenclature of cfDNA was used both for nuclear cfDNA (n-cfDNA) and mt-cfDNA, and each type of cfDNA showing different structures and functions. In Zhong’s study, mt-cfDNA was detectable in plasma and serum samples from healthy subjects and patients with diabetes. Moreover, the authors also detected a mitochondrial mutation commonly found in patients with maternally inherited diabetes in both samples of patients with diabetes. Please see the recent review by Bronkhorst et al. [13] for more details about the different nomenclature.

**Figure 1:**
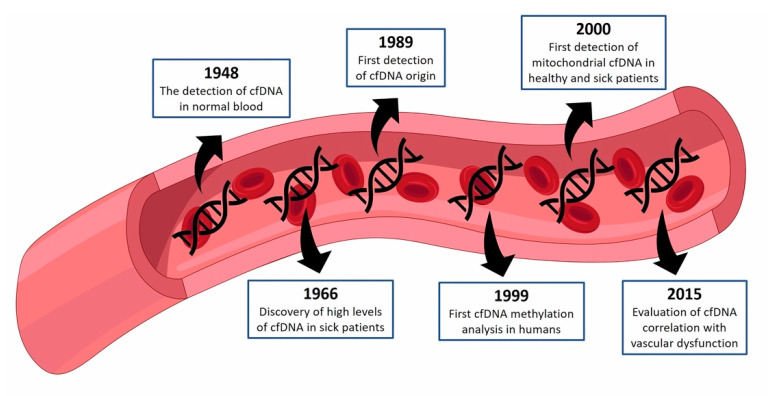
Chronological summary of cfDNA. The timeline shows the main cfDNA discoveries, from the first report in 1948 until the first correlation of cfDNA with vascular dysfunction in 2015. cfDNA: circulating cell-free DNA.

Lately, a growing number of publications show the scientific interest of cfDNA in clinical applications. Quantification of cfDNA concentration as a tool for noninvasive diagnosis and monitoring was performed in several acute and chronic disorders and different conditions like tissue damage, cell death, and turnover [14,15,16,17,18,19]. Figure 1 provides a timeline of the main discoveries concerning cfDNA.

## 2. cfDNA—Source and Mechanism of Release

**Figure 2:**
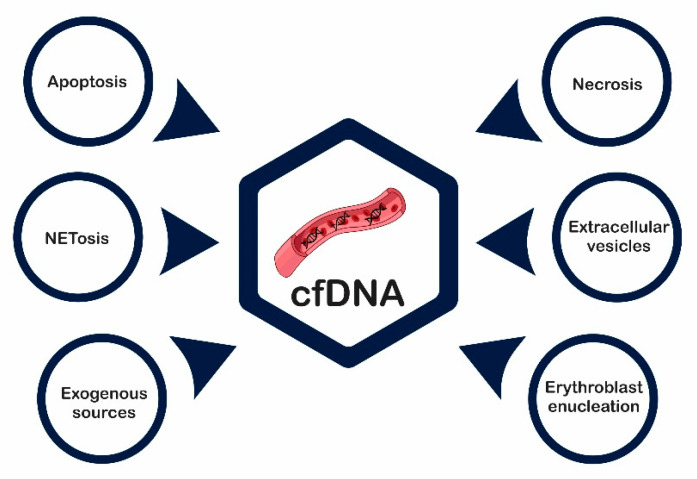
cfDNA sources. cfDNA is released into the human blood circulation by normal cells and cells involved with pathologic processes, including cell death. cfDNA: circulating cell-free DNA.

cfDNA can be found in many body fluids, both in physiological conditions as well as in pathological disorders. Different mechanisms allow the release of DNA fragments from the intracellular to the extracellular compartment. In healthy and diseased (benign and malignant) individuals, the release processes of DNA into the human blood circulation can originate from: (1) necrosis, (2) apoptosis, (3) active DNA release, and (4) exogenous sources (Figure 2).

Necrosis is the premature death of cells caused by an injury. Noxious stimuli due to external factors, such as infections (bacteria, viruses, fungi, parasites), toxins, hypoxia, and extreme environmental conditions (heat, radiation, or ultraviolet irradiation), lead to irreversible injury of the tissue and cell death by necrosis. When cells die by necrosis, they exhibit nuclear chromatin clumping and nonspecific digestion patterns, organelle and cell swelling, plasma membrane disintegration, and other cellular components release [20,21]. Jahr et al. [22] proposed that longer cfDNA fragments (i.e., >10 kb) are often observed in cancer patients, indicating an origin from necrosis. Many studies, had reported that fragments released from necrotic cells are often much larger than apoptotic DNA fragments because, despite necrosis occurs more rapidly than apoptosis, the removal of necrotic cells is slower [23,24,25]. In vitro assays, performed by Choi et al. [26], showed that the release of necrotic DNA fragments is phagocytic clearance dependent.

On the other hand, apoptosis is programmed cell death. There are a wide variety of stimuli and conditions, both physiological and pathological, that can trigger apoptosis. The mechanisms of apoptosis are complex involving an energy-dependent cascade of molecular events. The apoptotic cells exhibit cellular shrinkage and pyknosis followed by fragmentation of the nucleus. The cells are smaller in size, the cytoplasm is dense, and the organelles are more tightly packed. Every organelle integrity is still maintained, and all of this is enclosed within an intact plasma membrane [18,19,25]. Many studies suggest that the bulk of cfDNA found in healthy and diseased individuals is released during apoptosis [24]. A hallmark of apoptosis is chromosomal DNA degradation, in large fragments (50–300 kb) and subsequently into multiples of nucleosome units (180–200 bp) via caspase-activated DNase (CAD) [22,27,28]. This evidence was founded through a ladder pattern in electrophoresis assay. The separation of extracted cfDNA displays fragment ladders that ranged from ~160 to 1000 bp. The size of these fragments is due to multiple DNA lengths in nucleosomes and predominantly corresponds to mono- and oligonucleosomes. This feature is characteristic of caspase-dependent cleavage by the apoptosis pathway [22,29,30]. The presence of apoptotic cells is short-lived due to highly efficient phagocytic clearance mechanisms orchestrated by a series of intercellular events coordinated by a complex signaling network [22,25].

In addition to necrotic or apoptotic cell death, the active DNA release is described as another cfDNA release mechanism. Recent in vitro cell culture studies have demonstrated the presence of cfDNA in culture medium at levels that, which do not correlate with the processes of apoptosis, necrosis, or DNA replication. Wang et al. [31] assessed the release pattern of cfDNA from breast cancer cell lines. Interestingly, they showed the cfDNA concentration did not correlate with the amount of apoptotic and necrotic cells. However, if more cells were in the G1 phase, more cfDNA was detected. Aucamp et al. [32] evaluated characteristics of the cfDNA released by eight different cell lines and concluded its active release levels correlate cell line’s growth rate and cancer status of the cell line through its dependence on glycolytic activity. Chen et al. [33] reported increases in the concentration of cfDNA derived from cancer cells cultured in vitro during cell proliferation, indicating that a significant fraction of cfDNA is derived from active cellular secretions. The authors assume that the release of this cfDNA into the blood could transfect and transform adjacent or distant normal cells. While the exact mechanisms involved in the active release of cfDNA remain unclear, cfDNA may be released because of genomic instability.

Genomic instability plays an essential role in DNA replication mechanisms, as demonstrated by Diamond et al. The authors identified that tumor-derived exosomes transfer DNA to dendritic cells and activate them [34]. This release occurred after DNA accumulation in the cytosol and was regulated by the expression of the three prime repair exonuclease 1 (TREX1) in the parent cells [35]. In this context, it is important to highlight the cytosolic compartment is virtually devoid of DNA in physiological conditions. However, several situations can increase levels of single and/or double-stranded DNA molecules as, for example, due to cell death pathways activation involving mtDNA release into the cytosol [36], mitotic defects [37], and genomic instability by exposure to DNA-damaging agents [35]. Furthermore, cytosolic DNA accumulation may happen by genetic defects affecting the expression or catalytic activity of nucleases involved in this cellular pathway [38]. In keeping with this, mutations in TREX1, RNASEH2A, RNASEH2A, and RNASEH2C can source cytosolic DNA accumulation [39].

In contrast to various forms of cell death, active DNA release occurs in viable cells. Currently, active DNA release may include NETs (neutrophil extracellular trap), exosomes, and erythroblast enucleation. NETs are networks of extracellular DNA associated with histones, elastase, myeloperoxidase, antimicrobial peptides, and granule proteins that are involved in the direct attack and killing of pathogens [40,41]. NETs are an important defense mechanism against bacterial, viral, fungal, and parasitic infections [42]. However, recent evidence has suggested that NETs may have a role in noninfectious diseases, including systemic lupus erythematosus [43], atherosclerosis [44,45], endothelial cell damage [46], vasculitis [47], trauma [48], thrombosis [49], cancer [50], sepsis [51,52], and in the inflammatory response [40,53,54]. These situations are especially ones in which high levels of cfDNA have been reported. Other studies also observed high levels of NETs after intense physical exercise and, consequently, high levels of cfDNA in the bloodstream [55,56].

NETs are formed via a novel type of active cell death called as NETosis (pathogen-induced cell death, including the release of NETs). NETosis is a dynamic process that results in the release of DNA from neutrophils in two forms: suicidal and vital NETosis. Although the “osis” term indicates the cell death after NETs release, in some cases, NETosis can induce a rapid and vital form to release NETs, in which the neutrophils can still perform their phagocytic functions [57,58].

The suicidal NETosis is a cell death program that occurs when pathogen agents activate neutrophils. This process leads to chromatin decondensation, cell and nuclear membranes lysis, and finally, the release of NETs [41,42]. Of note, suicidal NETosis can take hours, even with high levels of antigen stimulation. On the other hand, the vital NETosis allows NET release through the blebbing of the nucleus, resulting in a DNA-filled vesicle that is exocytosed, leaving the plasma membrane intact without neutrophil death. Vital NETosis involves vesicular trafficking of DNA from within the nucleus to the extracellular space, contributing to cfDNA in circulation [59]. These anuclear cytoplasts formed that is capable of tracking and engulfing living bacteria. Its rapid formation and release can be completed in a matter of minutes [58].

Although NETosis was first described in neutrophils, similar processes have been described in other immune cells, including eosinophils, monocytes, and B cells, mast cells, basophils, and macrophages [54], which are collectively referred to as “ETosis”.

Another form of active release of cfDNA includes DNA fragments associated with extracellular vesicles, such as exosomes and microvesicles [1]. Although the release of apoptotic bodies during apoptosis has been long known [60], the fact that also perfectly healthy cells shed vesicles from their plasma membrane has only recently become appreciated [61,62].

Exosomes are intraluminal microvesicles that fusion with the plasma membrane and can be secreted by cells. Recently, Raposo et al. [63] reviewed exosome biogenesis. This cellular process begins with the invagination of the cellular plasma membrane, creating an early endosome. Then the Invagination of the plasma membrane of the early endosome produces a multivesicular body with cytosolic cellular components. The exosome is represented by small vesicles of different sizes into the multivesicular body and is released occurs by fusion multivesicular endosome with the plasma membrane. Another multivesicular endosome may fuse with lysosomes. The point of divergence between these types of multivesicular endosomes is drawn at early endosomes, but the existence of distinct early endosomes feeding into these two pathways cannot be excluded. The mechanism for intercellular communication involves the intercellular transfer of extracellular vesicles. Deficiencies in our knowledge of the molecular mechanisms for extracellular vesicle formation and lack of methods to interfere with the packaging of cargo or with vesicle release, however, still hamper identification of their physiological relevance in vivo.

The exosomes can be composed of proteins, lipids, mRNA, microRNA, or cfDNA [64,65,66]. The cfDNA are carried both on the surface and in the lumen [67] of the microvesicles measuring 30–100 nm [65,68]. Zocco et al. [69] and Lázaro-Ibáñez et al. [70] identified that most of the DNA associated with extracellular vesicles was located on the outside or surface of these extracellular vesicles. The content of the DNA of extracellular vesicles is quite diverse and heterogeneous, which would demonstrate vesicle subpopulations with different origins [70]. Recently, it has been demonstrated that microvesicles have functional and biological properties related to cellular communication, lateral transfer of material, immune system modulation, and cellular homeostasis maintenance [64,71,72].

An additional mechanism of active DNA release includes the nuclei expulsion from erythroblasts. Mature erythrocytes do not contain nuclei to optimize the unique function of ensuring proper oxygen delivery to the tissues [73]. Erythroblast enucleation is a stage of complex erythrocytes maturation process to produce a highly functional specialized cell [74]. At the end of erythroid precursors differentiation, among the changes occurring in this stage, cell cycle arrest, chromatin condensation, and nuclear polarization are essential for enucleation [75,76,77]. Then, erythroblasts lose all their organelles and expel their nuclei due to a process dependent on adhesion protein reorganization across the plasma membrane and macrophage interactions [78,79]. Kawane et al. [80] proposed that DNase II from macrophage lysosomes is responsible for DNA digestion and nuclei expulsion of precursor erythroid cells. Therefore, erythroblast enucleation serves primarily as a source of cfDNA in the bloodstream. LAM et al. [81] demonstrated that hematopoietic cells could contribute significantly to cfDNA origin. This cfDNA release has been directly associated with the increased erythropoietic activity of the bone marrow.

Additionally, cfDNA can also be released by exogenous sources. There is evidence that beyond the endogenous sources, foreign cfDNA from exogenous sources can be released into the bloodstream [82,83,84,85,86,87,88]. While endogenous cfDNAs originate from the cells of the organism of itself, exogenous cfDNAs are generally come from the host–microbiome [89], from different infectious agents (bacterial and viral) [90,91] and infestations (parasites) [85], as well as from the ingested food of the host organism [84]. Moreover, fetal cfDNA released into the maternal circulation [92] and cfDNA from organ transplantation [93] should also be considered.

## 3. cfDNA—Molecular Features

The pathophysiological importance of cfDNA is also related to its molecular characteristics. The cfDNA integrity (size) and its genetic and epigenetic profile and plasma concentration depend on its release mechanisms [29,94,95]. Furthermore, cfDNA includes n-cfDNA (nuclear) or mt-cfDNA (mitochondrial), with both types exhibiting different structural characteristics that potentially reveal different forms of biological stability [96]. The n-cfDNA are fragments of coding or non-coding genomic DNA. Among the coding and non-coding, n-cfDNA investigated, the housekeeping genes and tissue-specific genes have been used to study coding n-cfDNA. Repetitive sequences such as ALU (a short-interspersed nucleic element) and long-interspersed nucleotide elements (LINE1) have been used to explore the non-coding n-cfDNA. Both ALU and LINE1 sequences are distributed throughout the genome. In recent years, both n-cfDNA and mt-cfDNA have been used to examine mutations, methylation, copy-number variations (CNVs), cfDNA composition, and cfDNA fragmentation [97,98,99,100,101].

Some molecular biology techniques have been used to analyze cfDNA from plasma samples, including fluorescence [15], polymerase chain reaction (PCR) [9,14], quantitative real-time PCR (RT-qPCR) [16,102,103,104,105,106,107,108,109,110], droplet digital PCR (ddPCR) [87,111,112,113,114,115,116], array [111,117] and sequencing [86,105,106,107,113,117,118,119,120,121,122,123,124,125,126,127]. cfDNA also can be converted by bisulfite or digested with methylation-sensitive restriction enzyme (MSRE) for methylation analysis. The choice of the method depends on the different purposes of cfDNA detection and what molecular features will be analyzed. Fluorescence and conventional PCR are outdated techniques for analyzing cfDNA. Current PCR-based approaches have a lower cost, are easier to perform. However, there are a limited number of genes to be analyzed at a time. These need to be predetermined and eventually have less sensitivity. The array methodology allows the representation of the methylation status of cfDNA, identification of single nucleotide polymorphism (SNP) or CNVs, and search specific regions of interest. Sequencing-based approaches are more flexible, can be used for wide genomic analysis, and detect unknown mutations in specific genes. Despite the high cost, sequencing-based approaches are becoming a more available option [128,129,130,131,132]. Altogether, all of these cfDNA analysis leads to a final problem: a lack of an absolute and precise quantity of cfDNA that could be widely used as a reference value for routine clinical diagnosis. Although most of the studies report different amounts of cfDNA between the disease situation versus the control condition, there is still a huge variability among laboratories. The authors refer the reader to previous publications for information about pre-analytical recommendations towards an international guideline for cfDNA analyses [133,134]. The several molecular features of cfDNA will be addressed below.

### 3.1. cfDNA Integrity

cfDNA integrity can be evaluated by its fragmentation level. High- and low-weight cfDNA molecules can be detected in different fluids. The differences in cfDNA fragments size can indicate their origin or their pathophysiological conditions on the body. Apoptotic cells produce DNA fragments of 180–200 base pairs (bp), whereas necrotic cells release higher molecular-weight DNA fragments of over 10 kbp in size [22,25,135]. 

The most common size of cfDNA found is ~166 bp and consists of a histone-complexed DNA, called nucleosome [136,137]. This is the main cfDNA size found in plasma from all kinds of health or disease subjects [29,138,139,140]. In healthy individuals, the cfDNA size has been described to vary mainly between 70 to 200 bp [141,142,143]. Evaluating the fetal cfDNA size, Chan et al. [144] identified that only 20% of plasma samples had fetal cfDNA fragments larger than 193 bp, while none fragments were above 313 bp. These fragments are shorter than maternal cfDNA, which has a maximum value of 798 bp. Mouliere et al. have shown that tumor-derived cfDNA is highly fragmented and mainly composed of fragments < 145 bp [145].

### 3.2. Genetic and Epigenetic Profile

cfDNA released on biological fluids contains the same genetic and epigenetic variations as nuclear and mitochondrial DNA from viable cells. These changes may be include copy-number variations (CNVs) [146,147], mutations [146,148], cfDNA composition [89] and methylation changes [149]. Thus, through the analyses of cfDNA is also possible to study the genetic profile of a patient.

### 3.3. Copy-Number Variations (CNVs)

CNVs are an important class of mutation contributing significantly to genome instability in several pathologies [150,151,152]. Large-scale genome studies have identified cfDNA CNVs across various types of cancer and demonstrated their potential as cancer biomarkers [153]. Li et al. demonstrated that CNVs might provide a measurable classifier for assessing clinical outcomes in advanced colorectal cancer patients [154]. Xia et al. performed whole-genome sequencing of urine in prostate cancer patients [155]. CNVs analysis detected genomic abnormalities, including AR amplification, TMPRSS2-ERG fusion, PTEN gene deletion, NOTCH1 locus amplification along with genomic amplifications in specific regions of the chromosomes 8, 9, 11, and 14, and deletions in specific regions of the chromosomes 4, 5, 7, 12, and 16. In addition, the study demonstrated the potential clinical utility of urine cfDNAs in predicting treatment response and monitoring disease progression. Kutilin et al. analyzed CNVs for 30 genes in patients with lung cancer [156]. CNVs were detected for genes responsible for apoptosis regulation (BAX, P53, and CASP3), proliferation (SOX2), DNA reparation (XRCC1), oxidative phosphorylation (HV2), EGFR signaling pathway (GRB2, SOS1, MAPK1, STAT1, and BRAF), and for mir3678. These data made it possible to detect new molecular genetic markers for predicting metastases in the lung and greater knowledge for tumor resistance to target therapy.

### 3.4. Mutations

Mutation is a permanent alteration of the nucleotide sequence that makes up a gene. Mutations range in size and can affect anywhere from a single DNA base pair to a large segment of a chromosome that includes multiple genes. The mutation analysis of cfDNA in specifics genes as KRAS, TP53, BRAF, epidermal growth factor receptor (EGFR), and adenomatous polyposis coli (APC) has been demonstrated great clinical relevance [157,158,159,160,161,162]. Once the mutation is detected in cfDNA, it enables noninvasive tumor diagnosis, suggested that blood cfDNA may be a promising tool in cancer screening with higher sensitivity and specificity [22,163,164]. Min et al. confirmed that the KRAS mutation identified from the colorectal cancer tumor tissue samples was consistently detected in the plasma cfDNA [165]. In addition, as several therapeutic agents in clinical trials target specific pathways, the identification of the mutation can provide the status of the patient’s tumor to predict response to treatment.

### 3.5. cfDNA Composition

Changes in the physiological state of the organism can also be identified throughout the nucleic acid composition of the cfDNA. Natalya Veiko’s group [166,167] showed that diseases could cause either GC-enrichment of the cfDNA pool or its oxidation. Their data showed cfDNA was GC-enriched in cases of atherosclerosis, heart attack, and rheumatic arthritis [168,169,170], while in cancer, the cfDNA was both GC-enriched and oxidized [167,171,172]. One of the main reasons for apoptotic cell death in oxidative stress is reflected by an increase in the oxidative modification of cellular DNA. When the DNA is released into circulation, it continues to bear these marks of oxidative stress, particularly increased levels of 8-oxo-dG, an oxidation marker [167].

Sergeeva et al. [95] demonstrated the effect of a GC-rich cfDNA or oxidized cfDNA as a stress signal for the cell signaling pathways. cfDNA GC-rich simultaneously activated NRF2/KEAP1 and NF-kB signaling pathways and increased gene expression of MAP3K1, MAP4K4, NF-kB1A, REL, IKBKB, RelA, NRFKB, NF-KB1, and NF-kB2.

Increased content of CG in cfDNA is recognized by cells. Ermakov et al. demonstrated that cfDNA GC-rich is a potent stimulatory effect on human peripheral blood lymphocytes [173]. Kostyuk et al. reported the activation of the signaling pathway depends on TLR9 and consequently causes the positive regulation of TLR9 and MyD88 expression [174]. Thus, the composition of cfDNA can significantly influence cellular functional activity.

### 3.6. Epigenetic

Changes in cfDNA epigenetic modifications may also suggest a disbalance on the body. Epigenetic modifications are heritable molecular events that affect gene expression without changing DNA sequences, including DNA methylation and histone modification. DNA methylation refers to the addition of methyl group to cytosine residues in DNA sequence, and it is the best-studied epigenetic event [175,176]. DNA methylation is essential for normal cellular development and plays an important role in epigenetic control of gene activity.

Simultaneously, two groups performed for the first time cfDNA methylation evaluation in humans. On one side, Esteller et al. detected aberrant promoter hypermethylation of tumor suppressor genes in cfDNA from non-small cell lung cancer patients, while Wong et al. detected aberrant methylation of the p16 gene in the plasma and serum of hepatocellular carcinoma patients [10,11]. Both showed for the first time that cfDNA with aberrant promoter methylation could be detected in peripheral circulation cancer patients.

Different methylation patterns at CpG sites can be used to identify the cfDNA origin tissue. One limitation of total cfDNA quantifications is in non-identification of the tissue origin. Assays with appropriate target genes and their epigenetic signature have been one of the main factors to achieving relevant and accurate clinical effects [144,177].

Moss et al. developed an approach for unbiased determination of the tissue origins of cfDNA using a reference methylation atlas of 25 human tissues and cell types [177]. The authors identified that plasma cfDNA from healthy subjects originates mainly from hematopoietic cells (32%), erythrocyte progenitor cells (30%), lymphocytes (12%), monocytes (11%), vascular endothelial cells (9%), and hepatocytes (1%). Taken together, Moss et al. provided a detailed description of the composition of cfDNA in healthy people and proposed a new platform easily adapted to study the cellular contributors to cfDNA in many settings in healthy and pathologic human tissue dynamics.

### 3.7. cfDNA Concentration

The elevated level of cfDNA can reflect a physiological process, e.g., physical exercise [178,179,180] and pregnant women [181] or pathological processes, such as inflammation, diabetes, tissue trauma, sepsis, myocardial infarction, and patients that received transplantations [14,87,143,181,182,183,184,185,186]. 

The total cfDNA level in cancer patients has a significant increase with a wide range (hundreds to thousands ng/mL in the blood) compared with the healthy controls (a relative level of 30 ng/mL and ranging between 0 and 100 ng/mL) [136,143]. Leng et al. reported the concentration of cfDNA in patients with non-small cell lung cancer was higher than in healthy controls [187]. Zill et al. performed analyses of cfDNA sequencing data of patients with late-stage cancers across >50 cancer types. They showed a marked variation in blood ctDNA levels among patients with different tumor types [188]. Bettegowda et al. described lower amounts of cfDNA in patients with benign lesions or with early-stage cancer compared to patients with advanced or metastatic tumors [105]. This finding suggested that level of ctDNA can be changed according to the stages of the disease. Prakash et al. have been reported significant differences in the concentration of cfDNA during a perioperative process in donors and recipients undergoing living donor liver transplantation [189].

Finally, the concentration of cfDNA has been reporting as a potential predictor for clinical outcome in patients with ovarian and lung cancer [190,191]. Therefore, patients with high baseline cfDNA concentration had a significantly worse disease and overall survival than those with lower concentrations.

### 3.8. mt-cfDNA

mt-cfDNA has been found in healthy subjects and patients with breast cancer and acute ischemic stroke [192,193]. Mt-cfDNA can also be released into blood circulation by mechanisms of apoptosis, necrosis, and active cellular secretion, as discussed above. Mt-cfDNA has been shown to be more fragmented than n-cfDNA, typically ranging between 30 and 80 bp with peaks in 42–60 bp [192,194]. This smaller size can be ascribed to the absence of nucleosome-associated histone proteins, which render mt-cfDNA exposed to enzymatic cleavage [194,195].

Although the first evidence of cfDNA in the bloodstream was identified in 1948, only 52 years later, Zhong et al. [12] reported the presence of mt-cfDNA in plasma and serum samples. In their study, mt-cfDNA was detectable in healthy and diabetic patients. Moreover, the authors also describe a mitochondrial mutation commonly found in patients with maternally inherited diabetes. Jiang et al. showed elevated amounts of mt-cfDNA in hepatocellular carcinoma patients [29]. Mehra et al. demonstrated that mt-cfDNA levels do not always correlate with n-cfDNA levels [94]. The averages quantification of mt-cfDNA was higher than n-cfDNA levels, providing potentially distinct information with different sensitivity levels. Furthermore, Pinti et al. reported the copies number of mt-cfDNA increase significantly after the fifth decade of life, reaching its maximum value in the ninth decade [96]. This increase has been associated with the elevation of several proinflammatory cytokines, such as TNF-α, IL-6, RANTES, and IL-1ra. Due to its unique characteristics, such as small size, simple characterization by sequencing, and greater abundance, the mt-cfDNA can be used as a more sensitive diagnostic tool than n-cfDNA [87,196]. Moreover, Ingelsson et al. and Itagaki et al. reported other functions for mt-cfDNA. Lymphocytes, monocytes, and neutrophils can rapidly eject mtDNA, as network filament structures perform an important role in antimicrobial defense [197,198].

## 4. Clinical Findings

In the last years, cfDNA both in plasma or serum has been studied as a potential biomarker and noninvasive screening tool for many diseases, especially solid tumors and fetal genetic abnormalities.

In 1965, Bendidch and colleagues [3] showed that cfDNA derived from neoplastic cells could be involved in metastases. Almost ten years later, it was demonstrated by the radioimmunoassay technique that half of the cancer patients had significantly higher levels of cfDNA compared to controls [5]. In addition, in the early 1970s, cfDNA was described in patients with autoimmune disease [4]. As previously mentioned, all these observations have highlighted the interest in its potential as a noninvasive prognostic and diagnostic biomarker for various diseases.

The development of new molecular techniques allowed the reproducible detection and identification of low levels of cfDNA from a background mixture signal, permitting the detection in other physiological conditions, most successfully the fetal-derived cfDNA during pregnancy [118], myocardial infarction [15], stroke severity [16] and also as a minimally invasive screening tool for many diseases, especially solid tumors and fetal genetic abnormalities.

One of the most significant discoveries for applying cfDNA was identifying fetal cfDNA in maternal blood [9], which enabled developing genetic tests in prenatal care. The origin of the fetal cfDNA found in maternal blood has been described from the placenta, fetal hematopoietic cells, and the fetus [102,103]. 

A multicenter study in the USA for prenatal screening for fetal aneuploidy tests using cfDNA had a lower false-positive rate in detecting trisomy 21 and 18, compared to the standard procedure [105]. Currently, the use of cfDNA is well established for fetal sex assessment, paternity testing, and detection of aneuploidies and trisomies [118,119,120], diagnosis of monogenic diseases [111,121], fetal sex determination for sex-linked disorders [117], and fetal RhD status [104]. The proportion of fetal cfDNA represents only a minor fraction of the total amount of cfDNA (3–25%) [122], and this concentration increase with gestational age, with a potential association with body mass index [87] and being better detected around the ten weeks of pregnancy [123].

Advances in eliminating the maternal background DNA and increasing the sensibility to detect small concentrations of fetal cfDNA were achieved in techniques related to DNA isolation, single-molecule amplification, and high-throughput sequencing, improving the accuracy and robustness of noninvasive prenatal testing for fetal cfDNA. Nowadays, fetal cfDNA is already used for clinical screening in fetal genetic abnormalities in high-risk pregnancies. Since 2011, cfDNA noninvasive prenatal tests have been commercially available to determine paternity, fetal sex and to identify abnormalities in chromosomes, especially for detecting the most prevalent chromosomal aneuploidies, such as Down syndrome [199,200].

Another area of huge interest is studying cfDNA in oncology, with many findings reporting an association between cfDNA and cancer. In 1977, Leon et al. reported a significantly elevated serum DNA level in cancer, demonstrating that the serum of cancer patients contains higher concentrations of cfDNA than those of healthy individuals [5]. However, the importance of cfDNA in clinical cancer research was recognized in 1994, when point mutations of the N-RAS gene were identified in cfDNA of patients with acute myelogenous leukemia and myelodysplastic syndrome [7]. The U.S. Food and Drug Administration (FDA) approved in 2016 the first liquid biopsy test for commercial use. This diagnostic test detects deletions in exon 19 and substitution mutations of exon 21 in the epidermal growth factor receptor (EGFR) gene to identify patients with metastatic non-small cell lung cancer (NSCLC) who would be eligible for treatment with erlotinib [201]. In 2020 FDA approved the first liquid biopsy NGS diagnostic tests for commercial use. These diagnostic tests identify mutations in different genes in patients eligible for specific treatments, including patients with breast cancer, NSCLC, and prostate cancer. The full list of approved nucleic acid tests, including cfDNA tests, can be viewed on the FDA website [202].

The fragment size and/or variations in the genetic abnormalities from circulating cfDNA can be identified and differentiated from normal cells, representing up to 1% of the total cfDNA [203]. Notably, cfDNA can also be detected in other biological materials, such as stool, urine, saliva, pleural fluid, and cerebrospinal fluid [204]. Several studies [203,204,205,206] described that liquid biopsy is cheaper and less invasive to the patient, establishing this test as a method to detect a tumor before the onset of clinical symptoms and identify drug resistance through the quantification of cfDNA.

Today, the potential of cfDNA is validated by many clinical studies [204,205,207], and recent publications have pointed technical advances in analyses and sequencing that have led to dramatic improvements to differentiation of cfDNA targets, such as mutations in tumor-suppressor genes, activated oncogenes, hypermethylation, or chromosomal disorders [208], thereby increasing the clinical utility of cfDNA as an oncogenic marker. This provides deeper insights into tumor development and response to different treatments in the face of cancer evolution.

The levels of cfDNA in the circulation of cancer patients have been related to disease stage, varying the percentage in localized tumors to metastatic tumors [105], to tumor burden, and to the aggressiveness potential of the disease [106]. Of interest, in clinical screening and follow-up, cfDNA was detected in early-stage cancers (breast, colorectal, lung, and ovarian) associated with disease progression and survival of cancer patients [107].

Recent publications describe the release of cfDNA in different types of cancer. In ovarian cancer, for example, there are considerably increased levels of cfDNA in diagnosed patients than in healthy individuals or in patients with benign ovarian diseases, with significantly decreased after surgery [209]. The same happens in breast cancer, prostate cancer, stomach cancer, lung cancer, and others [207]; in all, high cfDNA levels were significantly associated with a higher tumor stage, which was correlated with worse survival. Besides the cfDNA concentration in plasma or serum, researchers also studied the methylation status of cfDNA in patients with hepatocellular carcinoma that may be involved in the inactivation of tumor suppressor genes [124]. It is clear to conclude that cfDNA has the potential to be considered as a biomarker of diagnosis and prognosis in cancer diagnosis, evidencing the potential to detect mutation and cellular abnormalities.

Since the discovery of donor-derived cfDNA (ddcfDNA) in organ recipients, by detecting Y-chromosome genes in the blood of sex-mismatched transplant recipients, the potential of ddcfDNA has been discussed as a cost-effective marker of rejection or as an indication of the health of the graft [14,108,109,112]. 

Interestingly, since ddcfDNA is a nonspecific organ or disease biomarker, it could be considered as a universal marker applicable for clinical monitoring of tissue injury in heart, lung, liver, and kidney allografts [14].

Publications reported [86,113], in heart transplantation, a percentage of ddcfDNA less than 1 % at stable patients and increased up to 5 % during a rejection episode. The same was observed in lung and renal transplantation [86,114]. In liver transplantation, a reduction of ddcfDNA was observed rapidly after ten days post-transplant, which remained stable in the absence of rejection. The level of ddcfDNA has been detached as a general biomarker of organ integrity, the severity of rejection [110,125], and as an early marker of rejection. The increased levels of ddcfDNA are associated with cellular rejection, as already seen in heart transplant [113], liver transplant [112], renal transplant [109], and lung transplant [86], while your rapid reduction post-transplant is considered a good prognostic indicator in grafts [208]. In the context of a bone marrow transplant, few studies report the relation of release ddcfDNA with cancer relapses and graft versus host disease [126].

Recently, Schütz et al. [115] studies showed that the ddcfDNA in blood samples increased on the first day of the transplant, associated with an ischemia and reperfusion injury, gradually decreasing to a relatively stable level, moreover, was observed relation between ddcfDNA and liver function, showing that ddcfDNA can have the same sensitivity to reflect graft damage. In renal transplant, the increased ddcfDNA in blood samples could discriminate active rejection from non-rejection, validating using ddcfDNA in the blood as an accurate marker of kidney injury/rejection [127]. Interestingly, ddcfDNA was found to be elevated up to 5 months before the biopsy-proven rejection event, suggesting a potential role in a heart transplant [125].

Another application of ddcfDNA has been the monitoring of drug immunosuppression dose effective to avoid toxicity and dose adjustment. In one study, the lower blood concentrations of ddcfDNA levels from liver transplant patients were associated with higher tacrolimus concentrations [116]. Kanzow et al. observed that the adjustment of tacrolimus dose from subtherapeutic to therapeutic levels was associated with a significant decrease in serum ddcfDNA fractions [210].

**Figure 3:**
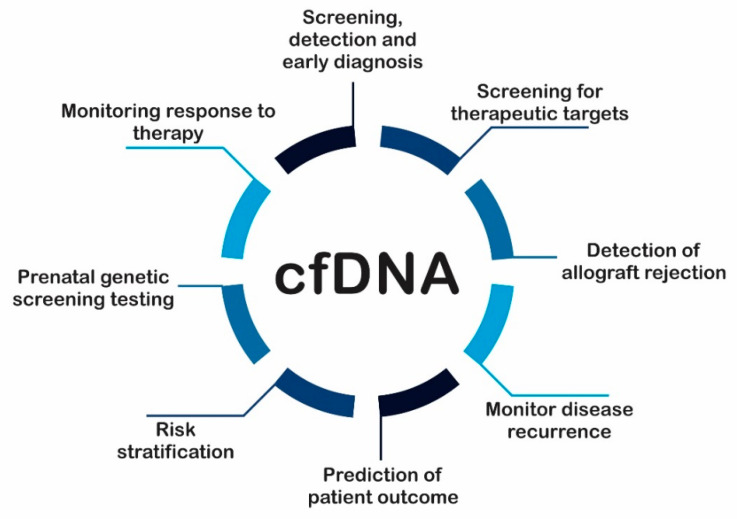
Potential clinical applications of cfDNA analysis. cfDNA can increase under abnormal conditions thus can be used as noninvasive biomarkers in several diseases and stages. cfDNA: circulating cell-free DNA.

CfDNA has already had a huge impact on prenatal medicine and could become, soon, an excellent tool in oncology, transplant medicine and also in conditions like cardiovascular diseases and sepsis. It is known that further studies should be conducted to understand the real role of cfDNA (Figure 3).

## 5. cfDNA as a Biomarker for Endothelial Dysfunction

Blood vessels are structurally divided into three layers from the most internal to the most external: intimate tunica, medium tunica, and tunica adventitia. The intimate tunica, also called endothelial layer (or endothelium), is basically composed of (i) endothelial cells, which are in direct contact with the blood, and (ii) the basement membrane, the layer that supports the endothelial cells [211].

**Figure 4:**
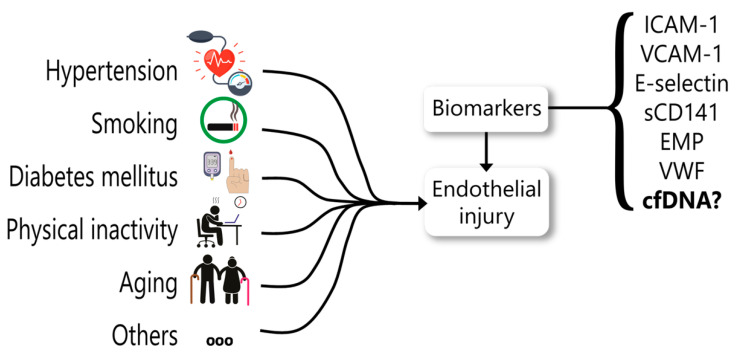
Circulating biomarkers proposed for endothelial dysfunction. Endothelial injury may be due to many conditions, including hypertension, diabetes mellitus, smoking, physical inactivity, aging, among others several clinical complications. Biomarkers are a valuable tool in clinical research and medical practice to identify an endothelial injury. ICAM-1: intercellular adhesion molecule 1; VCAM-1: vascular cell adhesion molecule 1; EMP: endothelial microparticles; VWF: von Willebrand factor; cfDNA: circulating cell-free DNA.

Initially, the endothelium was considered as a passive barrier between blood and the other vascular layers. Far beyond this concept, it has been described that the endothelium also plays a fundamental role in body homeostasis, being recognized as an important autocrine, paracrine, and endocrine organ [212,213]. Thus, endothelial injury is the bridge between risk factors and their consequences, such as infarction and stroke. Therefore, the search for biomarkers of endothelial dysfunction is extremely important (Figure 4).

Biomarkers of endothelial injury are valuable tools in clinical practice. Circulating biomarkers of endothelial injury have the advantage related to the simplicity of the procedures and to the fact that venous blood collection is commonplace in clinical practice. Endothelial dysfunction biomarkers have already been reviewed elsewhere [214]. Adhesion molecules, such as E-selectin, intercellular adhesion molecule 1 (ICAM-1), and vascular cell adhesion molecule 1 (VCAM-1), as well as other molecules involved in the coagulation pathway, such as von Willebrand factor (vWF) and soluble thrombomodulin (sCD141), are among the most studied [215].

The first study linking cfDNA and vascular dysfunction dates from 2015 [17]. McCarthy et al. hypothesized that the mt-cfDNA was elevated in hypertension, activating Toll-like receptor-9 (TLR9) and leading to endothelial dysfunction. Using spontaneous hypertensive rats (SHR), the authors first showed elevated levels of mt-cfDNA in male SHR, but not in female animals. Wistar Kyoto normotensive rats had increased systolic blood pressure within three days of i.p. injection of mt-cfDNA, but not with n-cfDNA. No changes in body mass, total heart mass, left ventricular mass, right ventricular mass, or spleen mass was reported. Regarding vascular dysfunction, mesenteric resistance arteries from mt-cfDNA-treated rats were less sensitive to acetylcholine (an endothelium-dependent vasodilator), but no difference was observed in the relaxation with the endothelium-independent vasodilator (the nitric oxide-donor, sodium nitroprusside). These effects were reversed by blocking the TLR9, thus demonstrating the involvement of the mt-cfDNA and the innate immune system pattern recognition receptor TLR9 in the pathogenesis of hypertension and endothelial dysfunction.

Coscas et al. in 2017 [216] studied the role of cfDNA in the initiation of vascular calcification. DNA structure is an important source of phosphates, and the poly-anionic nature of cfDNA may cause it to strongly interact with cationic calcium phosphate. Using human aorta samples that displayed early stages of atheroma, the authors identified cfDNA as a potential bed for calcium phosphate precipitation and hydroxyapatite crystallization through colocalization of cfDNA with sites of calcification. In addition, using the rat model of vascular calcification (intra-aortic infusions of cfDNA and elastase), the author observed that cfDNA was able to penetrate into the arterial wall and induce vascular wall calcification. The authors conclude that cfDNA could represent one type of mechanism able to initiate calcium phosphate precipitation and calcium phosphate apatite crystal formation. However, this mechanism does not exclude other possible processes, such as those involving microvesicle formation by injured cells [217].

Also, in 2017, Paunel-Gorgulu et al. [218] studied the role of cfDNA as an endothelial damage marker in patients after cardiac surgery with cardiopulmonary bypass (CPB). cfDNA levels were measured in the patient’s plasma at the time of admission and after surgery. Plasma cfDNA levels strongly increased after surgery in patients undergoing cardiac surgery both with short-time CPB (<100 min) or long-time CPB (>100 min). Although this initial increase was similar between groups, cfDNA levels remained significantly elevated until day 5 only in the long-time CPB (>100 min) patients. In addition, real-time PCR analyses revealed that mtDNA levels were significantly increased only in patients with long-time CPB (>100 min), indicating a more severe disease and more prone to systemic inflammation. In this study, the authors also measured sCD141 (soluble thrombomodulin) and ICAM-1 as molecular markers of endothelial cell injury. Both sCD141 and ICAM-1 levels were higher in patients with long-time CPB (>100 min). However, only sCD141 levels positively correlated with cfDNA levels.

The authors concluded that cfDNA represents an early biomarker for CPB-induced inflammation and a potential mediator of endothelial damage after cardiac surgery with prolonged bypass duration. The study also supported that cfDNA may potentialize inflammation, amplifying NETosis by an independent mechanism of endosomal TLR9 and ROS.

Recently, Yang et al. [219] proposed a blood test based on cfDNA that can predict the likelihood of a diabetes patient developing a microvascular complication. The author investigated the hydroxymethylation profile on cfDNA from patients with developed vascular complications versus those who did not yet manifest signs of vascular complications. cfDNA from 62 patients were sequenced, and for each gene, the extent of hydroxymethylation between patients with vascular complications and patients without was matched. This comparison showed almost 135 genes implicated in insulin resistance or inflammation with significantly different patterns of hydroxymethylation between groups. Among these genes, a specific selection of a 16-gene detection model showed superiority over commonly used clinical variables, including diabetic duration, body mass index, and endothelial growth factor receptor (eGFR). Similarly, a 13-gene detection model outperformed those clinical variables in terms of detection accuracy for distinguishing patients with single complications from those with multiple ones.

In conclusion, the authors showed that the hydroxymethylation profile of cfDNA might be in the future proven as a convenient and noninvasive marker for diabetes-induced vascular complications, with the potential to complement other conventional clinical variables or risk factors for disease monitoring. Although promising, the study lacks a cause-and-effect relationship between the epigenetics changes the vascular complications.

## 6. Conclusions

In this review, we approached the origin of cfDNA until its clinical use with a deeper focus as a biomarker for endothelial injury. We showed that cfDNA is already at an advanced stage for clinical use in prenatal tests, organ transplant rejection, and cancer, where it also received a more specific abbreviation, *t*-cfDNA. In the cardiovascular system, the studies are quite new, and although interesting, stronger evidence is still needed. Among all the characteristics of the cfDNA, there is evidence indicating that the methylation pattern of cfDNA is the most promising tool for this molecule to advance as a biomarker of tissue-specific injury, as in the case of endothelial injury.

However, some drawbacks in cfDNA methodologies should be overcome before its recommendation as a biomarker in the clinical setting. Due to its low abundance in circulation, some pre-analytical steps, such as plasma extraction, should be standardized. Some of the studies mentioned in this review have used homemade protocols, while others may have used extraction by magnetic beads, spin columns, vacuum columns, or even so any combinations from these methods. cfDNA is also rapidly cleared from the blood (from several minutes), leading to some negative data in the literature.

Finally, and perhaps the most important, is the method to detect cfDNA. The most used ones are using fluorescent probes (such as PicoGreen or SYBR Gold), RT–qPCR, and more recently, next-generation sequence (NGS) and ddPCR. The fluorescent techniques have less sensitivity and are unspecific, allowing to detection of any DNA molecule present in the circulation without any discrimination. These methods usually lead to overestimation of total cfDNA concentration. On the other hand, PCR is the most common. Although this technique overcame the problem of specificity by using specific primers to detect specific DNA sequences, is raised another problem that was the variability among the primers used by different groups.

Altogether, all of these steps in the cfDNA analysis lead to a final problem: a lack of an absolute and precise quantity of cfDNA that could be widely used as a reference value for routine clinical diagnosis. Although most studies report different amounts of cfDNA between the disease situation versus de control condition, there is still a huge variability among laboratories.
